# *Bifidobacterium longum* subsp. *infantis* ATCC 15697 and Goat Milk Oligosaccharides Show Synergism In Vitro as Anti-Infectives against *Campylobacter jejuni*

**DOI:** 10.3390/foods9030348

**Published:** 2020-03-17

**Authors:** Erinn M. Quinn, Helen Slattery, Dan Walsh, Lokesh Joshi, Rita M. Hickey

**Affiliations:** 1 Teagasc Food Research Centre, Moorepark, Fermoy, P61 C996 Co. Cork, Ireland; erinn.quinn@teagasc.ie (E.M.Q.); helen.slattery@teagasc.ie (H.S.); 2 Advanced Glycoscience Research Cluster, National Centre for Biomedical Engineering Science, National University of Ireland Galway, H91 TK33 Galway, Ireland; lokesh.joshi@nuigalway.ie; 3 Department of Microbiology, University College Cork, T12YT20 Co. Cork, Ireland; DWalsh@ucc.ie

**Keywords:** *Bifidobacterium*, *Campylobacter*, adhesion, milk oligosaccharides, synbiotics, HT-29 cells

## Abstract

Bifidobacteria are known to inhibit, compete with and displace the adhesion of pathogens to human intestinal cells. Previously, we demonstrated that goat milk oligosaccharides (GMO) increased the attachment of *Bifidobacterium longum* subsp. *infantis* ATCC 15697 to intestinal cells in vitro. In this study, we aimed to exploit this effect as a mechanism for inhibiting pathogen association with intestinal cells. We examined the synergistic effect of GMO-treated *B. infantis* on preventing the attachment of a highly invasive strain of *Campylobacter jejuni* to intestinal HT-29 cells. The combination decreased the adherence of *C. jejuni* to the HT-29 cells by an average of 42% compared to the control (non-GMO treated *B. infantis*). Increasing the incubation time of the GMO with the *Bifidobacterium* strain resulted in the strain metabolizing the GMO, correlating with a subsequent 104% increase in growth over a 24 h period when compared to the control. Metabolite analysis in the 24 h period also revealed increased production of acetate, lactate, formate and ethanol by GMO-treated *B. infantis*. Statistically significant changes in the GMO profile were also demonstrated over the 24 h period, indicating that the strain was digesting certain structures within the pool such as lactose, lacto-*N*-neotetraose, lacto-*N*-neohexaose 3′-sialyllactose, 6′-sialyllactose, sialyllacto-*N*-neotetraose c and disialyllactose. It may be that early exposure to GMO modulates the adhesion of *B. infantis* while carbohydrate utilisation becomes more important after the bacteria have transiently colonised the host cells in adequate numbers. This study builds a strong case for the use of synbiotics that incorporate oligosaccharides sourced from goat′s milk and probiotic bifidobacteria in functional foods, particularly considering the growing popularity of formulas based on goat milk.

## 1. Introduction

Bifidobacteria are considered one of the first colonisers of the human gastrointestinal (GI) tract and are suggested to confer positive health outcomes to the host, explaining their prevalence as probiotics [1]. These bacteria are particularly effective at protecting against infectious diseases, regulating immune responses and exerting effects against conditions ranging from irritable bowel syndrome, allergic diseases, ulcerative colitis, and immunoglobulin E associated diseases, to atopic dermatitis [2]. In terms of protecting against infection, bifidobacteria can operate through strain-specific antagonistic means for the competitive exclusion of pathogens [3]. A strain of *B. breve* was found to inhibit the growth of enterotoxigenic (ETEC) and enteropathogenic (EPEC) *Escherichia coli*, while other species were found to inhibit intestinal colonisation of pathogens such as *Salmonella*, *Shigella*, *Listeria monocytogenes*, and *Clostridium difficile* [4,5,6]. Microbe-associated molecular patterns (MAMPs) are recognized by the host′s intestinal pattern recognition receptors (PRRs), and these interactions play key roles in the association of pathogens with the intestinal epithelia. Probiotics also express molecular patterns which can recognize the same trans-membrane receptors as the pathogens, thus blocking the sites for pathogenic contact by competitive exclusion and, in some cases, displacing already-attached pathogens [7]. However, the health benefits associated with bifidobacteria are reliant on such strains colonising the host in sufficient numbers [8]. The important step in microbial colonisation of the intestinal epithelium is the attachment of bacterial surface lectins to intestinal sugar structures. Recent studies have suggested that milk oligosaccharides may enhance the specific ability of bifidobacteria to attach to the GI epithelium [9,10,11]. Indeed, our group investigated the ability of goat milk oligosaccharides (GMO) to increase the attachment of *Bifidobacterium longum* subsp. *infantis* ATCC 15697 to HT-29 cells. Exposure of the strain to the GMO resulted in an 8.3-fold increase in its adhesion to the intestinal cells [11].

As well as aiding the colonisation of bifidobacteria, GMO can also act as a prebiotic. The prebiotic potential of GMO recovered from whey has been identified in vitro where significant growth of *Bifidobacterium* spp. was observed on isolated GMO [12]. Oligosaccharide-enriched fractions prepared from both stage one and stage two goats′ milk-based infant formula were also recently shown to significantly enhance the growth of bifidobacteria in vitro and reduce the adhesion of *E. coli* NCTC 10418 and *Salmonella enterica* subsp. *enterica* serovar Typhimurium to Caco-2 cells [13], possibly by acting as analogues of epithelial receptors on the gut cells [14]. In vivo studies have also indicated that ingestion of GMO by mice during gestation and lactation increased the relative abundance of bifidobacteria in the colon of their pups at weaning [15]. These studies suggest that a combination of both a probiotic and a prebiotic (GMO) could provide a synergistic effect and may be an effective strategy to enhance the persistence and metabolic activity of specific beneficial bifidobacterial strains. Therefore, the aim of the current study is to examine such a synbiotic combination in vitro and determine if GMO have the potential to increase the competitiveness and metabolic activity of *B. infantis* in the intestinal tract. The ability of the synbiotic to competitively exclude an invasive strain of *Campylobacter jejuni* to intestinal cells is first examined. Fermentation of the GMO by the *B. infantis* strain is then investigated through growth studies, metabolite analysis and oligosaccharide depletion assays.

## 2. Materials and Methods

### 2.1. Oligosaccharides Standards

The oligosaccharide standards; 2′-Fucosyllactose (2′FL), Lacto-*N*-tetraose (LNT), 3′-Sialyllactose (3′SL), 6′-Sialyllactose (6′SL), Disialyllactose (DSL), Lacto-*N*-hexaose (LNH), *N*-Acetylneuraminic acid (Sialic Acid), LS-tetrasaccharide c (LSTc), Lacto-*N*-neotetraose (LNnT) and Lacto-*N*-neohexaose (LNnH) were purchased from Carbosynth Ltd. (Berkshire, UK) and lactose was obtained from VWR (Dublin, Ireland).

### 2.2. Isolation of Goat Milk Oligosaccharides

Goat milk oligosaccharides (GMO) were isolated and characterized as previously described [11]. In brief, mature milk from goats was kindly donated by Ardsallagh Goat Farm (Carrigtwohill, Co. Cork) and stored at −80 °C on arrival. To generate low molecular weight fractions, the milk was initially defatted and de-caseinated as per Quinn et al. [11]. Large peptides and whey proteins were removed by ultrafiltration and the permeates were freeze-dried as previously described [11]. To separate lactose from the oligosaccharides, a BioGel P2 size exclusion column (Bio-rad, Deeside, UK) was employed and the fractions collected were analysed for lactose, 3-SL and 6-SL using high pH anion exchange chromatography with pulsed amperometric detection (HPAEC-PAD) as detailed below, and the protein/peptide concentration was determined by the Bradford assay [16]. Peptide-free and low-trace lactose (<80 mg/L) fractions were pooled and freeze-dried to give an oligosaccharide-enriched fraction.

### 2.3. Milk Oligosaccharide Analysis

Oligosaccharide analysis of the pooled GMO was performed as previously described [11]. Oligosaccharide-enriched fractions were diluted in water and analysed in order to quantify levels of lactose, 2′FL, LNT, 3′SL, 6′SL, DSL, LNH, Sialic Acid, LSTc, LNnT and LNnH using a Dionex ICS-3000 Series system (Dionex Corporation, Sunnyvale, CA, USA) equipped with an electrochemical detector.

### 2.4. Bifidobacterium longum subsp. infantis Culture Conditions

*Bifidobacterium longum* subsp. *infantis* ATCC^®^ 15697™ (*B. infantis*) was obtained from the American Type Culture Collection (ATCC, Middlesex, UK). Bacterial cultures were maintained as previously described [10,11] The strain was stored in deMan Rogosa Sharpe (MRS) (Difco, Sparks, MD,, USA) broth containing 50% glycerol at −80 °C. The strain was cultured twice in MRS media supplemented with L-cysteine (0.05% *w/v*) (Merck, Dannstadt, Germany) prior to use, and was routinely grown overnight at 37 °C under anaerobic conditions generated using an Anaerocult A system (Merck).

### 2.5. Campylobacter jejuni Culture Conditions

*Campylobacter jejuni* 81–176 (*C. jejuni*) is a well-characterized, mobile flagellated invasive strain which has been used in many previous studies [17,18]. The pathogen was stored in Mueller–Hinton broth (Oxoid, Ireland c/o Fannin Healthcare, Dublin, Ireland) containing 50% glycerol at −80 °C and cultured directly from storage onto Mueller–Hinton agar plates. The pathogen was grown under microaerophilic conditions generated using CampyGen gas packs (Oxoid), for 48 h at 37 °C. Prior to pathogen inhibition assays, *C. jejuni* 81–176 was grown on Mueller–Hinton agar and then transferred to biphasic media in 25 cm^2^ tissue culture flasks (Corning, NY, USA) consisting of Mueller–Hinton agar supplemented with *Campylobacter* selective supplement (Skirrow), (Oxoid) and 6 mL of McCoy′s 5A media (Merck) supplemented with 2% FBS. The flask was then incubated for 24 h under microaerophilic conditions at 37 °C.

### 2.6. Exposure of B. infantis to Goat Milk Oligosaccharides

Exposure of the bacteria to GMO was performed as previously described [11]. A final concentration of 5 mg/mL of GMO was used reflecting the concentration of oligosaccharides present in mature human milk [11,19,20,21,22,23,24,25,26,27]. Bacterial suspensions were then incubated for 1 h at 37 °C under anaerobic conditions. Following this, bacteria were harvested by centrifugation (3850× *g*, 5 min), the supernatants removed, and pellets were washed three times in phosphate-buffered saline (PBS) and then re-suspended in non-supplemented McCoy′s media prior to use in the adhesion assays.

### 2.7. Mammalian Cell Culture Conditions

HT-29 cells were grown as previously described [11] and maintained in McCoy′s 5A modified medium (Merck) supplemented with 10% fetal bovine serum (FBS) using 75 cm^2^ tissue culture flasks incubated at 37 °C in 5% CO_2_ in a humidified atmosphere. Once the cells were nearing confluency (approximately 80%–90%), they were passaged into 48 well tissue culture plates (Sarstedt Ltd., Wexford, Ireland) at a density of 1 × 10^5^ cells/mL between passages 15–21. The cells were then used once fully confluent (approximately 2 × 10^6^ cells/well). The media was changed every other day and supplemented with 2% FBS 24 h prior to use.

### 2.8. Adhesion Assays with B. infantis

Adhesion assays with *B. infantis* were performed as previously described [11]. HT-29 cells were washed twice with PBS, and 250 µL of the bacteria and media suspensions were added to the wells, corresponding to approximately 40 bacterial cells per human cell. Bacterial cells were incubated with the HT-29 cells after which they were washed with PBS to remove non-adherent bacteria. HT-29 cells were then lysed and the lysates were serially diluted and enumerated by spot-plating on MRS plates to enumerate bacterial colony forming units (CFU). The adhesion of the bacteria was determined as the percentage of original inoculum that attached, thus accounting for variations in the starting inoculum. Percentage adhesion = (CFU/mL of recovered adherent bacteria/CFU/mL of inoculum) × 100. Experiments were performed in triplicate on three separate occasions.

### 2.9. Anti-Infective Assays and Exclusion Assay

Anti-infective assays were performed as previously described [18] with minor modifications. In brief, *C. jejuni* was incubated in the absence and presence of GMO (5 mg/mL) at a final OD_600nm_ of 0.3 (approximately 5.14 × 10^8^ CFU/mL) in McCoys′s media and incubated under microaerophilic conditions for 1 h at 37 °C; 250 µL of the mix was then applied to three wells containing HT-29 cells, and allowed to incubate for 3 h, after which the eukaryotic cells were washed five times with PBS, lysed with 250 µL 0.1% Triton X-100 (Merck) in PBS and spread plated onto Mueller–Hinton agar plates and incubated under microaerophilic conditions for 72 h at 37 °C to enumerate CFU. For exclusion assays, exposure of *B. infantis* to 5 mg/mL GMO was performed as described above, and this suspension was subsequently incubated with the HT-29 cells for 2 h. A non-supplemented control was also included. Non-adherent bacteria were removed from the cells as described above, after which the cell line was challenged with *C. jejuni*. To do this, the pathogen was harvested from the biphasic medium, washed twice in non-supplemented McCoy′s, and diluted to an OD_600nm_ of 0.3. From this suspension, 250 μL was then added to each well, and cells were incubated under anaerobic conditions for 3 h at 37 °C. Cells were then washed five times with PBS, lysed with 0.1% Triton X-100 (Merck) in PBS and plated onto supplemented Mueller–Hinton agar. Mueller–Hinton plates were incubated under microaerophilic conditions for 72 h at 37 °C, after which bacterial CFU were enumerated. The assays were performed in triplicate on three separate occasions. The exclusion of *C. jejuni* was determined as the average CFU/mL of recovered adherent bacteria. The percentage decrease in *C. jejuni* adhesion was calculated as the difference in *C. jejuni* CFU/mL between non-supplemented and GMO-supplemented bifidobacteria.

### 2.10. Effect of GMO on the Growth of B. infantis

*B. infantis* was grown in the absence and presence of 5 mg/mL GMO over a 72 h period under adhesion assay conditions. Aliquots of 150 μL of the bacterial suspensions were added to the individual wells of a 96 well microtiter plate. Other controls included a control containing no bacteria, and bacteria grown in deMan Rogosa Sharpe (MRS) (Difco, Sparks, MD, USA) broth supplemented with L-cysteine (0.05% *w/v*) (Merck). These experiments were performed in a concept 400 anaerobic chamber (Baker, ME, USA) and bacterial growth was monitored by determining OD_600nm_ using a Synergy-HT multidetector microplate reader driven by Gen5 reader control and data analysis software (BioTek Instruments Inc. Bedfordshire UK) at 0, 12, 24, 48 and 72 h. The microtitre plate was automatically shaken for 30 s prior to each measurement to achieve a homogenous suspension. The results are represented as the average OD_600nm_ of triplicate experiments performed on three separate occasions. The percentage increase in growth of *B. infantis* was calculated as the difference in OD_600nm_ between non-supplemented and GMO-supplemented bifidobacteria.

### 2.11. GMO Consumption by B. infantis

*B. infantis* was grown overnight under the optimal conditions outlined above and then re-suspended in McCoy′s media at an OD_600nm_ of 0.25 with a final oligosaccharide concentration of 5 mg/mL. A one milli-litre aliquot of this cell suspension was then dispensed into a sterile Eppendorf (Merck) after 0 h and 24 h of growth. A negative control containing no bacteria was also included. These solutions were then centrifuged for 5 min (3850× g) and the supernatants collected. The process was repeated a total of three times to ensure bacteria were not present in the supernatant. The sample was also treated with ultraviolet light for 30 min in a laminar flow hood to ensure no further metabolic activity occurred., 2′FL, LNT, 3′SL, 6′SL, DSL, LNH, Sialic Acid, LSTc, LNnT, and LNnH were quantified using HPAEC-PAD analyses to quantify oligosaccharide levels before and after 24 h of fermentation using a Dionex ICS-3000 Series system (Dionex Corporation). Reductions in the areas of the peaks after immediate exposure and over the 24 h period were calculated through comparative analysis as per Lane et al. [28]. These experiments were performed on three separate occasions.

### 2.12. B. infantis Metabolite Analysis

Supernatants collected for HPAEC-PAD analysis at 0 and 24 h were also assessed for changes in metabolite production using high-pressure liquid chromatography. Metabolic end products, lactate, acetate, formate and ethanol were measured using an Agilent 1200 HPLC system (Agilent Technologies, Santa Clara, CA, USA) with a refractive index detector. Metabolite peaks and concentrations were identified and calculated based on known metabolite retention times and standard solutions at known concentrations. A negative control of non-supplemented media was also included. A REZEX 8 m 8%H, organic acid column (300 × 7.8 mM Phenomenex, CA, USA) was used and the elution was performed for 25 min with a 0.01 M H_2_SO_4_ solution at a constant flow rate of 0.6 mL/min and a temperature of 65 °C. The standard solutions were prepared as per Table 1. These experiments were performed in triplicate. The ratios of acetate to lactate, acetate to formate, and lactate to ethanol were also calculated.

### 2.13. Statistical Analysis

Graphs were drawn using Microsoft Excel. The results are presented as the mean ± standard deviations of replicate experiments, and the unpaired Student *t*-test was used to determine statistically significant results. For all experiments, *p* < 0.05 was considered significant.

## 3. Results and Discussion

### 3.1. Characterisation of the Goat Milk Oligosaccharides

The batch of goat′s milk used in this study was previously characterised in terms of its oligosaccharide content [29]. Forty-one oligosaccharide structures were identified including both neutral and acidic structures, of which 64% of the acidic fraction were Neu5Gc linked [29]. In this study, we isolated oligosaccharide from the same batch of goats milk and quantified some of the major structures, which included LNnT, LNnH, 3′-SL, 6′-SL, LSTc and DSL (Table 2). In terms of the difference between human and animal milk composition, type I oligosaccharides predominate in human milk [27], while type II oligosaccharides are either exclusive or predominate over type I structures in animal milk. Regarding human milk oligosaccharide (HMO) structures, 70% are fucosylated, with lacto-*N*-biose (type I) structures (Gal(b1–3)GlcNAc) predominating over structures containing the *N*-acetyllactosamine (type II) (Gal(b1–4)GlcNAc) [29]. In contrast, animal-derived oligosaccharides are predominantly sialylated, containing *N*-acetylneuraminic acid (Neu5Ac) and/or *N*-glycolylneuraminic acid (Neu5Gc) [30,31]. However, it is important to note that the oligosaccharide yield obtained from animal milk is much lower than that of human milk. While changes in GMO composition occur across lactation [24], previous studies have indicated that a number of oligosaccharides, such as 3′ and 6′ sialylactose, β3′-galactosyllactose, β6′-galactosyllactose, 2′-fucosyllactose, Lactose-*N*-hexaose, 6′-*N*-acetylneuraminyllactose and 3′-*N*-acetylneuraminyllactose are present in both human and goat milk [32,33,34,35]. In addition, a recent study by Leong et al. 2019 [13] investigated the presence of naturally occurring oligosaccharides in commercial goats′ milk-based stage one and stage two infant formulas and their prebiotic properties. Fourteen quantifiable oligosaccharides in goats′ milk-based infant formula were detectable by LC/MS, indicating that, structurally, GMO may represent an alternative to the human milk derivatives where breastfeeding is not possible. In addition, high purity and recovery of GMO consisting of 67.6% acidic and 34.4% neutral oligosaccharides have been demonstrated [36], indicating that potentially viable commercial production methods may be available in the not too distant future.

### 3.2. Combined Effect of GMO and B. infantis on C. jejuni Adhesion

**Figure 1:**
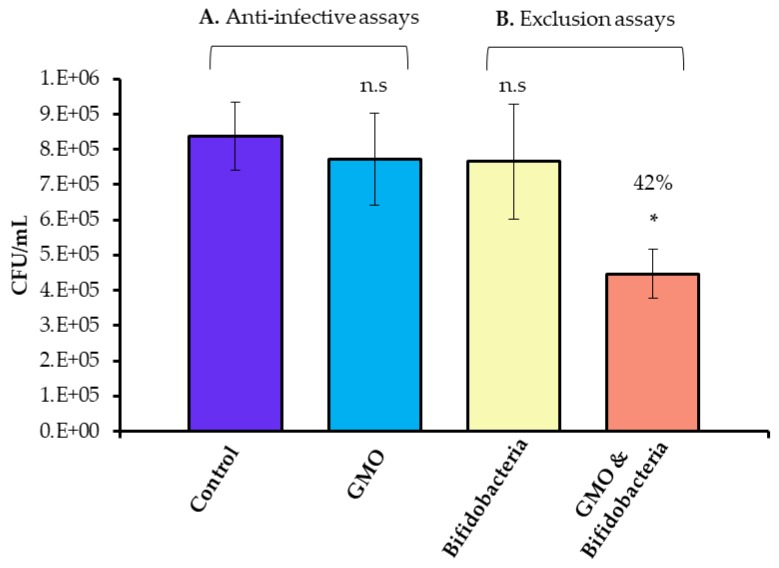
Anti-infective assays (**A**) demonstrating *Campylobacter jejuni* 81–176 adhesion in the absence and presence of GMO, and (**B**) competitive exclusion assays demonstrating *Campylobacter jejuni* 81–176 adhesion to HT-29 cells following pre-treatment of the HT-29 cells with *Bifidobacterium longum* subsp. *infantis* 15697 (yellow) and *Bifidobacterium longum* subsp. *infantis* 15697 pre-treated with GMO (orange). Results demonstrate the average colony forming units (CFU)/mL of adherent *Campylobacter jejuni* 81–176 of one representative triplicate experiment, with error bars representing standard deviation. The unpaired non-parametric t-test was used, *: *p*-value: < 0.05, n.s: not significant.

Probiotic bacteria, such as *Lactobacillus acidophilus* UO 001 and *Lactobacillus gasseri* UO 002, are known to inhibit the growth of *Campylobacter* without interfering with the normal microbiota of the gastrointestinal tract, suggesting other probiotic bacteria may have similar effects [37,38]. Murine fecal microbiota transplantation treatment was shown to alleviate intestinal and systemic immune responses in *C. jejuni*-infected mice harbouring a human gut microbiota. These with mice displayed higher numbers of lactobacilli and bifidobacteria, further suggesting the beneficial effects of probiotics against pathogen colonisation [39]. However, a prerequisite for survival in the intestinal tract is the ability of probiotic strains to transiently adhere efficiently to the intestinal mucosa. Probiotic and pathogenic strains have been suggested to share similarities in terms of their surface adhesins and, thus, may compete for adhesion sites [3,40]. Previously, we demonstrated an increase in *B. infantis* adherence to HT-29 cells after pre-exposure to GMO [11]. The HT-29 cells are extensively used as a model of the gastrointestinal tract, particularly as in vitro intestinal models of bacterial colonisation [41,42]. These cells exhibit classical characteristics that model small intestinal absorptive epithelial cells upon reaching confluence [43] and are a useful indicator of the structural landscape of the intestinal epithelium [44]. In the current study, we also employed HT-29 cells and hypothesised that the increase in *B. infantis* adhesion following GMO treatment may provide a protective effect against *C. jejuni* colonisation of HT-29 cells. We pre-treated *B. infantis* with GMO and observed an average increase in adhesion of 4.4 fold, demonstrating a clear increase in adhesion potential over three biological replicate experiments performed in triplicate (Figure S1). Following this, anti-infective assays were conducted to investigate if GMO alone could prevent *C. jejuni* colonisation. Overall, an average inoculum of 5.14 × 10^8^ CFU/mL was applied to the HT-29 cells, of which 1.35 × 10^6^ CFU/mL of *C. jejuni* were demonstrated to adhere. The percentage of *C. jejuni* from the original inoculum which adhered to the HT-29 cells was 0.26% ± 0.05. Notably, both GMO alone and *B. infantis* alone had no protective effects against *C. jejuni* colonisation (*p*-value: > 0.5) (Figure 1). Exclusion assays assessed the ability of *B. infantis* alone and pre-treated with GMO to prevent attachment and invasion of *C. jejuni* to HT-29 cells. The assays revealed a prophylactic protective effect following prior treatment of *B. infantis* with GMO (Figure 1) with an average significant decrease ranging from 42%–46% in *C. jejuni* adherence observed over triplicate experiments performed on three separate occasions. Interestingly, the non-supplemented *B. infantis* control demonstrated no protective effect against *C. jejuni* colonisation. This may suggest that a critical population level of attached bifidobacteria is required to result in the competitive exclusion of a pathogen. In contrast to the results demonstrated here, a previous study in mice with *L. johnsonii* indicated no reduction in lower intestinal *C. jejuni* colonisation; however, a suppressed intestinal and systemic pro-inflammatory and enhanced anti-inflammatory immune response were both observed, indicating other potential benefits of the use of probiotics on *C. jejuni* infection [45]. Notably, no statistically significant increase in *C. jejuni* growth in the presence of GMO was observed under anti-infective and exclusion assay conditions.

The results presented here are particularly significant as *Campylobacter* is one of four key global causes of diarrhoeal diseases and is considered to be the most common bacterial cause of human gastroenteritis in the world [46] *C. jejuni*, in particular, poses a great risk worldwide due to its associated diarrhoeal disease and the risk of development of severe secondary diseases, such as irritable bowel syndrome and Guillain–Barré syndrome post-infection [17,47]. A number of studies have suggested that probiotic bacteria may inhibit pathogens through competitive exclusion to pathogen adhesion sites and nutrients [48]. Commercial broiler chickens are a major reservoir for *C. jejuni* and consumption can lead to human infection [49]. However, swine and cattle can also act as zoonotic vectors in addition to ingestion of contaminated surface waters [50,51]. Notably, strains such as *Lactobacillus* spp., i.e., *acidophilus*, *casei*, *crispatus*, *gasseri*, *helveticus*, *pentosus*, *plantarum*, *rhamnosus*, and *salivarius* have been suggested to exhibit anti-*Campylobacter* activities in vitro and in vivo [49]. In addition, Dec et al. [52], in screening *Lactobacillus* isolates for anti-*Campylobacter* activity, selected seven *Lactobacillus* isolates with potential applications in reducing *Campylobacter* spp. in chickens, which may have potential to prevent infections in both birds and humans [52]. In vivo studies in poultry using synbiotic combinations of *Bifidobacterium longum* PCB 133 and galactooligosaccharides in animal feed have been shown to reduce *C. jejuni* infection, demonstrating the potential to reduce transmission along the food chain, which is of fundamental importance for the safety of poultry meat consumers [53]. Microencapsulated *Bifidobacterium longum* PCB133 and xylooligosaccharides (XOS), when combined, have also been shown to have a synbiotic effect on improving the safety of poultry meat by protecting against *C. jejuni* infection (*p*-value: < 0.01) at the beginning of life, while the microbiota is still developing [54]. Additionally, in vivo studies in chicks have demonstrated the ability of three chicken commensal isolates to induce a 1–2 log reduction in *Campylobacter* numbers. However, isolates were only capable of reducing *Campylobacter* levels in one out of three trials. Notably, follow–up experiments demonstrated *Lactobacillus salivarius* subsp. *salicinius*, in combination with 0.04% mannan oligosaccharides resulted in a 3-log decrease of cecal *Campylobacter* suggesting that, similar to the current study, the use of synbiotic combinations may be more effective at preventing *Campylobacter* colonisation when compared to a commercial strain alone [55]. The cumulative prebiotic and colonisation-promoting effect of the GMO on the *B. infantis* strain in the current study further supports the use of such synbiotics in foods aimed at preventing infection.

### 3.3. Prebiotic Effects of GMO

**Figure 2:**
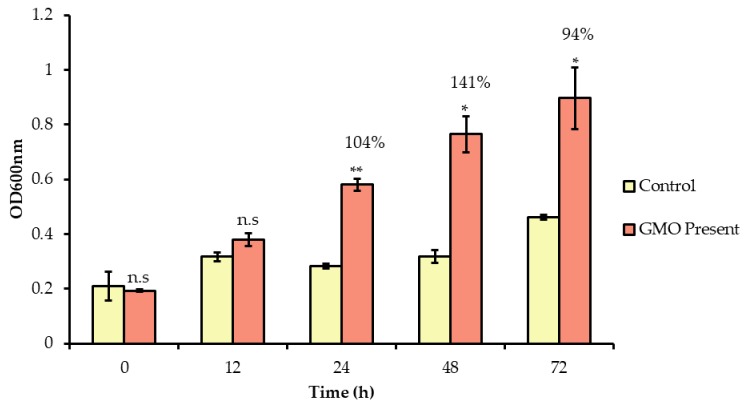
Growth of *B. longum* subsp. *infantis* in McCoy′s media supplemented with GMO over 72 h. Optical density readings were taken at time 0, 12, 24, 48 and 72 h. Results are represented as the average of three biological replicates, with error bars representing standard deviation. The unpaired non-parametric t-test was used, *: *p*-value: < 0.05, ** *p*-value: < 0.005, n.s: not significant.

The ability of GMO to stimulate the growth of *B. infantis* was determined using growth curves (Figure 2). When GMO was incubated with *B. infantis* following the pre-treatment period, an average increase in growth of 104% was observed in the presence of GMO at 24 h. No significant increases in the growth of *B. infantis* occurred during earlier incubation periods (≤12 h). Thus, it is unlikely that this growth contributed to the observed increase in bifidobacterial adhesion to the HT-29 cells after the 1 h exposure. 

Previous studies have demonstrated statistically significant increases in *Bifidobacterium* and *Bacteroides* growth following incubation with GMO [12]. Similarly, GMO has also been shown to increase levels of bifidobacteria using in vitro fermentation models and in in vivo mouse trials [15], [39]. The prebiotic effect observed here is not surprising given that *B. infantis* ATCC 15697 is particularly adept at the utilising human milk glycans due to the presence of a 43 kb gene cluster responsible for their transport and utilization [56,57,58,59,60,61,62,63,64]. Indeed, *Bifidobacterium longum* subsp. *infantis* has been described as the “champion colonizer of the infant gut” and is unique among gut bacteria in its prodigious capacity to digest and consume milk oligosaccharide structures [58,59,60]. Moreover, a number of bifidobacterial strains have the ability to utilize milk oligosaccharides as substrates for growth [65]. In vitro fermentation of human milk oligosaccharides by different bifidobacterial strains including *B. longum* subsp. *infantis* ATCC 15697, *B. longum* ATCC 15707, *B. breve* ATCC 27539, *B. adolescentis* ATCC 15703 and *B. bifidum* ATCC 29521 has been previously demonstrated [66].

**Figure 3:**
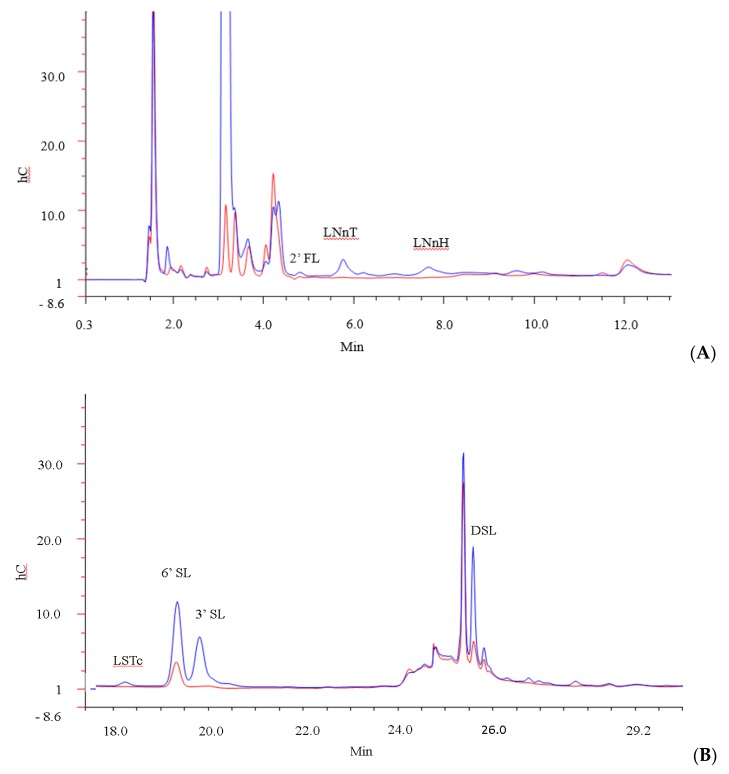
GMO profile of (**A**) high molecular weight oligosaccharides and (**B**) low molecular weight oligosaccharides after 0 h (blue) and 24 h (red) incubation with *B. infantis*.

The ability of *B. infantis* ATCC 15697 to use multiple specific carbohydrate structures (2′FL, LNT, 3′SL, 6′SL, DSL, LNH, Sialic Acid, LSTc, LNnT and LNnH) within the pool was also investigated by generating HPAEC-PAD profiles of the oligosaccharides in the media before and after 24 h of bacterial growth (Figure 3). Notably, before the incubation period, a total of 24 peaks were detected in the GMO by the method used, of which six (3′SL, 6′SL, DSL, LSTc, LNnT and LNnH) were identified. Following 24 h incubation with *B. infantis,* 19 peaks were detected, of which 11 unidentified peaks were found in trace amounts that were not detected in the starting material. 2′FL, LNT, LNH, and free sialic acid were not detected at either time point. Overall, our results demonstrated that the strain was capable of utilizing multiple structures. For instance, 3′ and 6′ SL were depleted by 94% and 71% respectively, while LNnT and LNnH, were 100% utilised. LSTc was depleted by 94%, DSL was depleted by 43.1%, and lactose was depleted by 52%. This may be expected as *B. longum* subsp. *longum* has been shown to utilize free LNnT, one of the dominant components of HMOs, [66,67]. In addition, *B. longum* subsp. *infantis* BRS8-2 and TPY1201 are known to degrade 2′FL, 3FL, 3′SL and LNnT, while *B. longum* subsp. *infantis* DSM 20088 has been shown to utilise 2′FL, 3FL, and LNnT [68]. Therefore, it is likely that the prebiotic effect associated with GMOs may extend to other strains.

The presence of bifidobacteria in the gut are known to influence the production of formate, acetate, ethanol and lactate [69] and gut homeostasis is achieved through their production [70]. The inhibition of gram-negative bacteria through the production of metabolites has been shown [71]. Acetate is the most prominent short chain fatty acids (SCFA) [72] and accounts for over half the total SCFA content in stools [73,74]. Acetate has been shown to improve protection against pathogen colonisation as it modulates the gut epithelium and induces anti-inflammatory and anti-apoptotic effects [75].

In this study, HPLC was implemented to determine the production of metabolites such as acetate, lactate, formate and ethanol by *B. infantis*, following growth on GMO (Table 3). The results indicated that GMO resulted in a 12-fold (*p*-value = 0.0009) higher concentration of acetic acid at 24 h in comparison to the non-supplemented control. Indeed, the concentration of lactic acid was 15-fold higher (*p*-value = 0.0019), while the concentration of formic acid was 8-fold (*p*-value = 0.0001) higher than the non-supplemented control at 24 h. Notably, ethanol was only detected in the GMO-supplemented sample at a concentration of 8mM at 24 h (Table 3). Notably, bifidobacterial strains can catabolize 2 moles of hexose resulting in the production of 2 moles of lactic acid and 3 moles of acetic acid using the fructose-6-phosphate phosphoketolase pathway, resulting in a theoretical yield of acetate:lactate ratio of 1.5:1 in hexose [76]. However, this ratio is rarely obtained and varies depending on the growth substrate and/or culture conditions [77]. For example, growth on substrates LNT, LNnT, inulin-type fructans and 2FL has been demonstrated to result in higher ratios of acetate to lactate [76,78,79]. Similarly, in this study, the acetate to lactate ratio of acetate to lactate produced by *B. infantis* was 3.3:1 following 24 h growth on GMO. Increased acetate production has been reported to occur in log-phase cells and relates to the phosphorolytic splitting of pyruvate derived from carbons four, five, and six of hexose and this could account for the additional levels of acetate we observed in this study [80]. The high ratio of acetate to lactate in substrated LNT and LNnT has been suggested to be in part a result of the deacetylation of the GlcNAc [76] and this, too, may have contributed the results found here. Notably, some bifidobacteria convert pyruvic acid into formic acid and ethanol rather than into lactic acid, thereby yielding an extra ATP [77]. In this study, we detected concentrations of ethanol (8.004 mM), formic acid (8.57 mM) and lactate acid (9.04 mM) after 24 h fermentation in GMO, which also may explain the lower than expected ratio of lactate to acetate. While bifidobacteria are incapable of producing butyrate, within the host, the production of acetate and lactate can be converted to butyrate through cross-feeding pathways via indigenous colonic species, such as *Faecalibacterium prausnitzii* (clostridial cluster IV) and *Anaerostipes, Eubacterium* and *Roseburia* species (clostridial cluster XIVa) [70,81,82,83,84] and, thus, in vivo, may have additional beneficial effects.

## 4. Conclusions

Initial exposure of bifidobacteria to GMO resulted in increased attachment to HT-29 cells and, in turn, had a prophylactic effect against *C. jejuni* attachment and invasion of intestinal cells in vitro. Protection against pathogen colonisation through competitive exclusion is a key benefit of the increasing bifidobacterial colonisation and could be exploited in order to deal with the rising numbers of *Campylobacter* infections. While other pathogens were not assessed in this study, there is potential that GMO treatment could provide overall resistance to pathogenic colonisation if used prophylactically as a synbiotic with bifidobacteria. Longer exposures of *B. infantis* to GMO increased the growth of the strain, highlighting its ability to adapt to environmental factors and promote its overall survival and colonisation in the GI tract, further highlighting the benefits of using synbiotic combinations. Overall, this study highlights the potential benefits in combining oligosaccharides from goat milk with select probiotic strains for promoting a healthy gut ecosystem, whilst protecting the host against pathogenic disease. Moreover, the use of such synbiotics is not limited to gastro-intestinal disorders, as low numbers of bifidobacteria have been associated with many other disorders. Such disorders include periodontal disease, rheumatoid arthritis, atherosclerosis, allergy, multi-organ failure, asthma and allergic diseases in addition to inflammatory bowel diseases, such as Crohn′s disease, irritable bowel syndrome and ulcerative colitis [85,86]. Synbiotics may, therefore, be more effective than either probiotics or prebiotics when used alone in the treatment and management of human health.

## Figures and Tables

**Table 1:** Volumes of standards used for metabolite analysis.

HPLC Standard	Per 100 mL	Molecular Weights
10 mM Lactic acid	0.09 g	90.08
10 mM Acetic acid	57 μL	60.05
10 mM Formic Acid	38 μL	46.03
10 mM Ethanol (99%)	58 μL	46.07

**Table 2:** Levels of different oligosaccharides present in goat milk ogliosaccharides (GMO) pool.

Oligosaccharide Structures	μg/mL
Lacto-*N*-neotetraose (LNnT)	3.4
Lacto-*N*-neohexaose (LNnH)	3
3′-Sialyllactose (3′SL)	32.83
6′-Sialyllactose (6′SL)	33.05
LS-tetrasaccharide c	0.94
Disialyllactose (DSL)	33.46

**Table 3:** Production of metabolites by *B. longum* subsp *infantis* American Type Culture Collection (ATCC) 15697 after 0 and 24 h incubation with GMO.

	Control		GMO	
Concentration mM	0 h	24 h	0 h	24 h
**Acetate**	ND	2.42 ^a^	0.07	30.14 ^a,b^
**Lactate**	ND	0.62 ^n.s^	ND	9.04 ^a,b^
**Formate**	0.81	1.13 ^a^	0.79 ^b^	8.57 ^a,b^
**Ethanol**	ND	ND	ND	8.00 ^a,b^

^a^ indicates significance in comparison to time 0 and 24 h within the one group, and ^b^ indicates significance in comparison to the non-supplemented control. ^n.s^ indicates not significant.

## Supplementary Materials

The following are available online at https://www.mdpi.com/2304-8158/9/3/348/s1, Figure S1: Adhesion of *B. longum* subsp. *infantis* ATCC 15697 to HT-29 cells following incubation with goat milk oligosaccharides.
