# Supplementary material for: Bifidobacterium longum subsp. infantis ATCC 15697 and Goat Milk Oligosaccharides Show Synergism In Vitro as Anti-Infectives against Campylobacter jejuni
Source: Foods. 2020 Mar 17;9(3):348. doi: 10.3390/foods9030348 (PMC7142803; doi:10.3390/foods9030348)
Supplement: Supplementary file 1 [file foods-09-00348-s001.pdf]

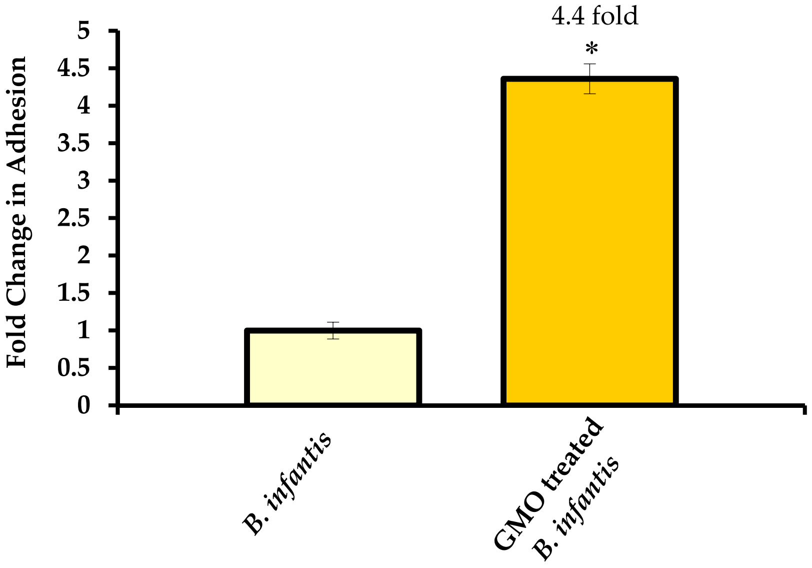

Figure S1. Adhesion of *B. longum subsp. infantis* ATCC 15697 to HT-29 cells following incubation with goat milk oligosaccharides. Results are represented as the average of triplicate experiments performed on three separate occasions and are presented as the percentage of adherent cells = [CFU/mL of recovered adherent bacteria ÷ CFU/mL of inoculum] × 100 and graphed as fold-change relative to percent adhesion of control, with error bars representing the standard deviation. The unpaired non-parametric t-test was used,* *p*-value: <0.05.
